# Involvement of PKR and RNase L in translational control and induction of apoptosis after Hepatitis C polyprotein expression from a Vaccinia virus recombinant

**DOI:** 10.1186/1743-422X-2-81

**Published:** 2005-09-12

**Authors:** Carmen E Gómez, Andrée Marie Vandermeeren, María Angel García, Elena Domingo-Gil, Mariano Esteban

**Affiliations:** 1 Department of Molecular and Cellular Biology, Centro Nacional de Biotecnología, CSIC, Campus Universidad Autónoma, 28049 Madrid, Spain

## Abstract

**Background:**

Hepatitis C virus (HCV) infection is of growing concern in public health with around 350 million chronically infected individuals worldwide. Although the IFN-α/rivabirin is the only approved therapy with 10–30% clinical efficacy, the protective molecular mechanism involved during the treatment is still unknown. To analyze the effect of HCV polyprotein expression on the antiviral response of the host, we developed a novel vaccinia virus (VV)-based delivery system (VT7-HCV7.9) where structural and nonstructural (except part of NS5B) proteins of HCV ORF from genotype 1b are efficiently expressed and produced, and timely regulated in mammalian cell lines.

**Results:**

Regulated transcript production and viral polypeptide processing was demonstrated in various cell lines infected with the recombinant VT7-HCV7.9, indicating that the cellular and viral proteolytic machineries are functional within these cells. The inducible expression of the HCV polyprotein by VV inhibits the synthesis of both host and viral proteins over the time and also induces apoptosis in HeLa and HepG2-infected cells. These effects occur accompanying with the phosphorylation of the translation initiation factor eIF-2α. In cells co-infected with VT7-HCV7.9 and a recombinant VV expressing the dominant negative eIF-2α-S51A mutant in the presence of the inductor isopropyl-thiogalactoside (IPTG), protein synthesis is rescued. The IFN-inducible protein kinase PKR is responsible for the translational block, as demonstrated with PKR-/- and PKR+/+ cell lines. However, apoptosis induced by VT7-HCV7.9 is mediated by the RNase L pathway, in a PKR-independent manner.

**Conclusion:**

These findings demonstrate the antiviral relevance of the proteins induced by interferon, PKR and RNase L during expression from a VV recombinant of the HCV polyprotein in human cell lines. HCV polyprotein expression caused a severe cytopathological effect in human cells as a result of inhibition of protein synthesis and apoptosis induction, triggered by the activation of the IFN-induced enzymes PKR and RNase L systems. Thus, the virus-cell system described here highlights the relevance of the IFN system as a protective mechanism against HCV infection.

## Background

The Hepatitis C virus (HCV) was identified as the causative agent for the majority of posttransfusion and sporadic non-A, and non-B hepatitis cases [1,2]. The World health organization (WHO) estimates that more than 3% of the world's population is infected with the virus. HCV belongs to the genus of Hepacivirus and is a member of the *Flaviviridae *family, along with Pestivirus and Flavivirus [3]. The HCV genome is a positively charged single stranded RNA molecule that includes two untranslated regions at the 5' and 3' ends, and a large open reading frame (ORF) encoding a 3010–3030 amino acid polyprotein that is co- and posttranslationally cleaved by cellular and viral proteases to produce mature structural (Core, E1, E2 and p7) and nonstructural (NS2, NS3, NS4A, NS4B, NS5A and NS5B) proteins [4,5]. One striking characteristic of HCV is its strong propensity to persist in the infected host, which often leads to severe liver damage, ranging from chronic hepatitis to liver cirrhosis and even hepatocellular carcinoma [6].

The IFN-α monotherapy became the mainstay for treatment of HCV infection until recently, when IFN-α/ribavirin, and pegylated IFN-α/ribavirin combination therapies became available [7]. The IFN-based regimens are still the only approved therapies for HCV [8]. Although the beneficial effect has been documented by numerous studies [9-11], only 10–40% of patients respond to treatment. The molecular mechanisms involved in protection during IFN therapy are not fully understood. Due to the clinical relevance of HCV infection and the differential responses of patients to IFN therapy, it is essential to investigate the molecular mechanisms involved in the sensitivity and resistance patterns of HCV infection in an appropriate model system.

In order to establish a robust *in vitro *infection model system for HCV, a variety of different approaches, mainly those based on infection with human patient sera of primary human liver cells or diverse cell lines of hepatic or lymphoid origin, have been explored [12,13]. Nonetheless, so far the success of these attempts has been limited due to the extremely low HCV replication levels that prevent detailed studies. The development of subgenomic HCV replicons that generates high-level replication of HCV RNAs in cell culture, has overcome this hurdle [14,15]. In spite of an efficient expression of the structural proteins and high levels of replication, it has not been possible to generate viral particles in cell cultures. Moreover, important information on the potential effect of the structural proteins on the host cell could not be obtained. An alternative approach has been viral delivery systems. In such systems, cells are transfected with a plasmid containing a cDNA clone under the control of a T7 promoter, and then infected with a virus that expresses T7 RNA polymerase. Although this approach has been met with some degree of success [16-18], it is limited by the efficiency with which the plasmid can be transfected into hosts cells. In the case of hepatocyte derived cell lines, the transfection efficiency is often rather low. This inefficiency could be overcome in certain cases, by using recombinant fowlpox viruses to deliver HCV minigenomes under the control of a T7 promoter into cells co-infected with an adenovirus expressing T7 RNA polymerase [19]. Although this system improved the efficiency of delivery, it was not possible to control HCV gene expression. Recently, a virus production system has been developed which is based on the transfection of the human hepatoma cell line Huh-7 with a genomic HCV RNA replicon derived from an individual with fulminant hepatitis [20]. The limited virus yields and virus spread of this cell culture system has been improved using a particular permissive cell line derived from Huh-7 designated Huh-7.5.1 [21]. This provides a significant advance in order to understand the biology of HCV infection in culture systems.

To characterize the antiviral response of the host during expression of the HCV polyprotein, we developed a novel poxvirus-based delivery system (VT7-HCV7.9), that is inducible and able to express structural and nonstructural (except part of NS5B) proteins of HCV ORF from genotype 1b in hepatic and non-hepatic mammalian cell lines. In this virus-cell system, we observed that HCV polyprotein expression controls cellular translation through eIF-2α-S51 phosphorylation, with involvement of the IFN-inducible double-stranded RNA-dependent protein kinase PKR. Moreover, in VT7-HCV7.9 infected cells, we found that HCV polyprotein expression brings about an apoptotic response through the activation of the RNase L pathway.

## Results

### Generation of a vaccinia virus recombinant expressing the near full-length HCV genome under regulation (VT7-HCV7.9)

**Figure 1:**
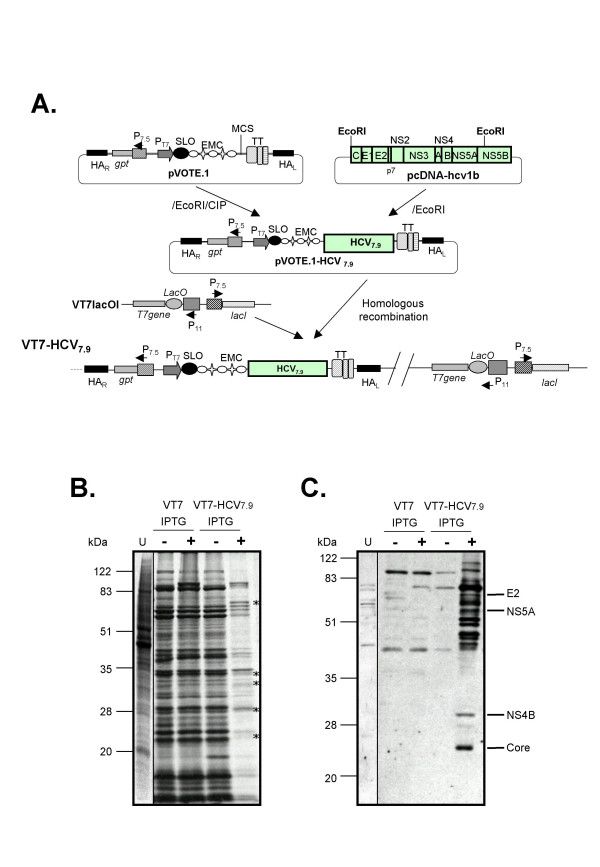
**Construction and characterization of the recombinant VT7-HCV7.9 virus**. **A**: Generation of recombinant VT7-HCV7.9. A 7.9 Kb DNA fragment containing the structural (C, E1, E2 and p7) and nonstructural (NS3, NS4A, NS4B, NS5A and the amino terminal region of NS5B) proteins of HCV from genotype 1b was cloned into a unique *EcoRI *restriction site of pVOTE.1 to make the plasmid transfer vector pVOTE.1-HCV7.9. BSC40 cells infected with the recombinant VT7lacOI (VT7), were transfected with the plasmid pVOTE.1-HCV7.9 as described in Materials and Methods to generate the recombinant VT7-HCV7.9. **B**: Expression of HCV inhibits protein synthesis in mammalian cells. Monolayers of BSC40 cells were infected at 5 PFU/cell with either the parental VT7 or the recombinant VT7-HCV7.9 viruses in the presence (+) or absence (-) of the inductor IPTG. Uninfected (U) and infected cells were metabolically labelled with ^35^S-Met-Cys Promix (100 μCi/mL) from 4 to 24 h.p.i. as described in Materials and Methods. Approximately 100 μg of total cell protein extracted from uninfected (U) and infected cells, was fractionated by SDS-PAGE followed by autoradiography. (*) represents new additional polypeptides corresponding to the HCV proteins. **C**: Inducible expression of HCV proteins by recombinant VT7-HCV7.9 virus. BSC40 cells were infected as described above. Total cell protein lysates from uninfected (U) and infected cells at 24 h.p.i. were analysed by Western blot using a human anti-HCV antibody from an infected patient. The protein band migration of Core, E2, NS4B and NS5A, as determined with specific antibodies, is indicated.

In order to study the effect of HCV gene expression on host cellular mechanisms, we developed a novel system based on a poxvirus vector that when induced, expresses the structural and nonstructural (except part of NS5B) proteins of HCV ORF from genotype 1b. Briefly, BSC40 cells infected with the recombinant VT7lacOI virus, that inducibly expresses the T7 RNA polymerase, were transfected with the plasmid transfer vector pVOTE.1-HCV7.9. This transfer vector directs the insertion of the HCV DNA fragment into the viral hemagglutinin (HA) locus under the transcriptional control of the T7 promoter, to generate the recombinant VT7-HCV7.9 (Figure 1A). Upon induction with IPTG, the T7 RNA polymerase is expressed which in turn, allows the transcription of HCV genes in VT7-HCV7.9 infected cells.

**Figure 2:**
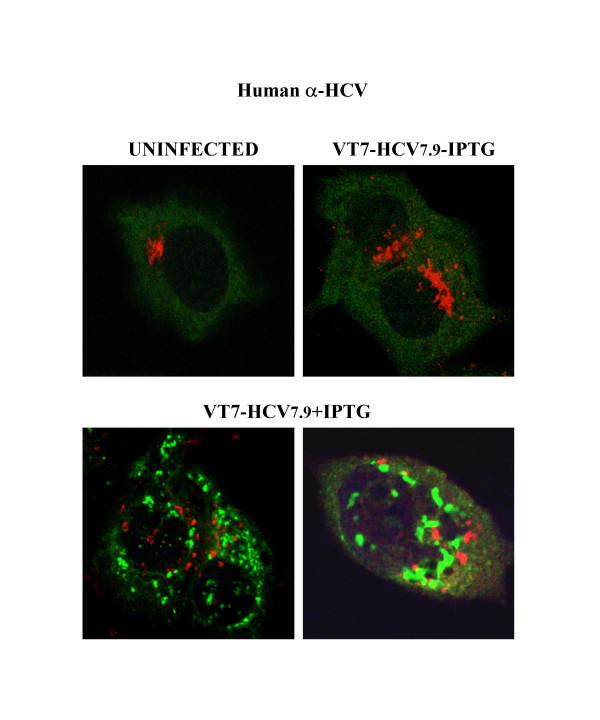
**Cellular localization of HCV proteins by immunofluorescence microscopy**. Subconfluent HeLa cells were infected at 5 PFU/cell with the recombinant VT7-HCV7.9 in the presence (+) or absence (-) of the inductor IPTG. At 16 h.p.i, cells were doubly labelled with polyclonal antibody anti-Gigantine to detect the Golgi complex (red) and a 1/200 dilution of serum from an HCV-infected patient (green) followed by the appropriate fluorescent secondary antibody and ToPro reagent.

To confirm expression of HCV proteins from the VV recombinant, we infected BSC40 cells with VT7-HCV7.9 and employed metabolic labelling, immunoblot and immunofluorescence microscopic analyses. Continuous metabolic labelling of BSC40 cells infected with VT7-HCV7.9 in the presence of IPTG, revealed by SDS-PAGE the synthesis of polypeptides not present in the absence of IPTG (Figure 1B, see new proteins denoted with asteriks). Significantly, in the presence of IPTG, overall protein synthesis was reduced in VT7-HCV7.9 infected cells when compared to protein synthesis in the absence of the inductor. This translational inhibitory effect was specific, since protein synthesis was not affected in cells infected with VT7, with or without IPTG (Figure 1B). The synthesis of HCV proteins in VT7-HCV7.9 infected cells was also documented by Western blot analysis, using sera from an HCV-infected patient. As shown in Figure 1C, HCV proteins of the expected size, for structural and nonstructural polypeptides, were detected only in VT7-HCV7.9 infected cells upon induction with IPTG. The size of specific HCV proteins was confirmed following reactivity with antibodies against Core, E2, NS4B and NS5A (not shown). A heterogeneous pattern of HCV-specific proteins was observed, perhaps as a result of different stages of proteolytic processing of the polyprotein. Confocal microscopy using sera from an infected patient revealed that the HCV proteins expressed in VT7-HCV7.9 infected cells upon induction with IPTG, formed large cytoplasmic aggregates and produced severe disruption of the golgi apparatus, a phenomenon not observed in cells infected in the absence of IPTG (Figure 2). The HCV proteins Core, E2, NS4B and NS5A were individually detected intracellularly with specific antibodies in VT7-HCV7.9 infected HeLa cells upon induction with IPTG (not shown).

The results of Figures 1, 2 reveal that the HCV ORF included in the recombinant VT7-HCV7.9 is efficiently transcribed during infection in the presence of IPTG, generating a viral polyprotein that is processed into mature structural and nonstructural HCV proteins, triggering disruption of the golgi apparatus.

### Expression of HCV polyprotein from VV inhibits the production of vaccinia virus

**Figure 3:**
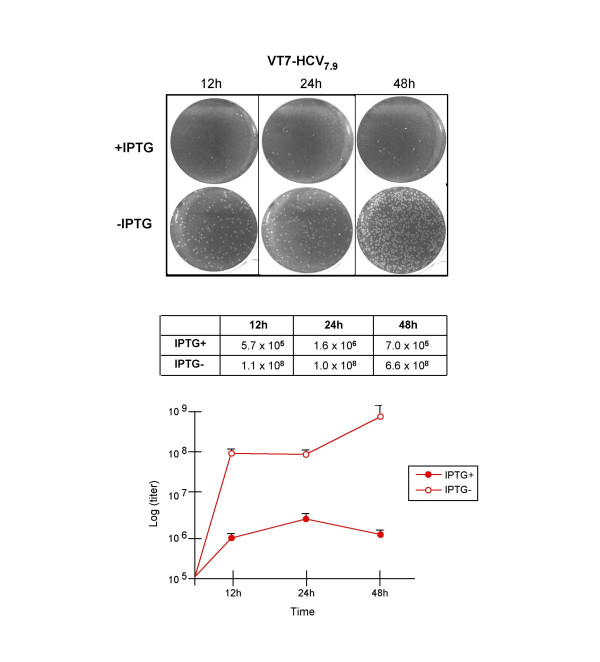
**Expression of HCV polyprotein inhibits the production of infectious VV**. BSC40 cells were infected at 5 PFU/cell with the recombinant VT7-HCV7.9 in the presence or absence of IPTG. After the indicated times postinfection the cells were collected, centrifuged and resuspended in 300 μL of DMEM. After three freeze-thawing cycles, followed by sonication, the cell extracts were titrated in BSC40 cells. The experiment was performed two times in duplicate. Means and standard deviations are shown.

To determine the impact of HCV gene expression on the replication of the recombinant VT7-HCV7.9 virus, we studied the production of infectious VV at 12, 24 and 48 h.p.i, in the presence or absence of the inductor IPTG. As demonstrated in Figure 3 by virus plaque formation and virus titration curves, the production of infectious VV was significantly reduced (over 2 logs) during HCV gene expression. These results reveal that expression of HCV impairs VV replication.

### Expression of HCV polyprotein from VV inhibits cellular and viral protein synthesis through eIF-2α phosphorylation

**Figure 4:**
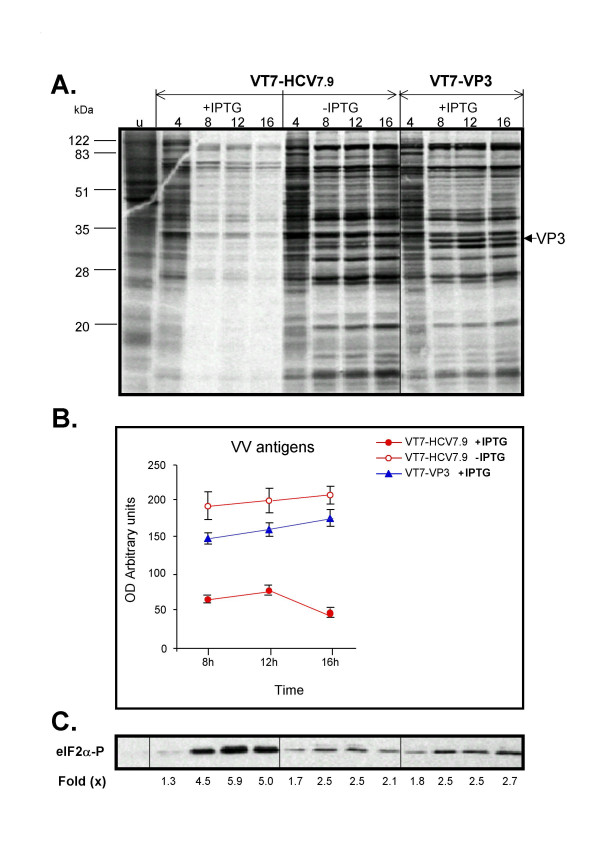
**Time-course analysis of cellular and viral protein synthesis in cells expressing HCV polyprotein**. **A**: BSC40 cells infected with the recombinant VT7-HCV7.9 virus in the presence (+) or absence (-) of IPTG were metabolically labelled with [^35^S] Met-Cys Promix (50 μCi/mL) at the indicated times (h.p.i) and analysed by SDS-PAGE (12%) and autoradiography. For comparative purposes, we included a similar inducible recombinant virus but expressing the IBDV mature structural capsid protein VP3 (VT7-VP3). **B**: Inhibition of VV proteins after expression of HCV. The levels of VV proteins were quantitated from autoradiograms using a BioRad GS700 image densitometer and computer software as suggested by the manufacturer. **C**: Immunoblot analysis of phospho-eIF-2α-S51 protein levels during the time-course of VT7-HCV7.9 infection. The number appearing in each lane represents the ratio of phospho-eIF-2α-S51 levels in infected cells compared to levels in uninfected cells.

Next, we determined the nature of the translational block in cells infected with VT7-HCV7.9 in the presence of IPTG. As a control, we included a recombinant VT7-VP3 inducibly expressing the IBDV capsid protein VP3. This virus was constructed similarly to VT7-HCV7.9, and expresses an mRNA encoding VP3 ORF from the vaccinia virus genome via T7 polymerase. Cells infected with VT7-HCV7.9, in the presence or absence of IPTG, were metabolically labelled for 30 min with ^35^S-Met-Cys Promix at 4, 8, 12 and 16 h.p.i., whole cell lysates fractionated by SDS-PAGE and the protein pattern examined by autoradiography. As shown in Figure 4, a clear reduction in cellular and viral protein synthesis was observed after 4 h.p.i in cells infected with the recombinant VT7-HCV7.9 virus in the presence of IPTG, in contrast with cells infected in the absence of the inductor, or in cells inducibly expressing the VP3 protein (Figure 4A). The protein levels were quantified by densitometry of the bands and are represented in Figure 4B. A strong decrease in protein synthesis becomes apparent by 8 h.p.i.

Phosphorylation of the α subunit of the eukaryotic translation initiation factor 2 (eIF-2) on serine 51 leads to the downregulation of translation initiation through a well-characterized mechanism involving inhibition of eIF-2B activity [22]. As such, we determined whether HCV polyprotein expression altered this initiation step. Thus, the levels of phospho-eIF-2α-S51 in VT7-HCV7.9 infected cells, in the presence or absence of IPTG, were determined by immunoblot analysis. The results obtained showed that expression of HCV is related to levels of eIF-2α-S51 phosphorylation over time, relative to non-induced VT7-HCV7.9 infected cells (Figure 4C). Similar levels of phosphorylation have been shown to cause growth inhibitory effects in yeast, as well as in mammalian cells [23]. The levels of phospho-eIF-2α-S51 in VT7-VP3 infected cells in the presence of IPTG at the assayed times, were similar to the levels obtained in uninduced VT7-HCV7.9 infected cultures (Figure 4C), and represent the values usually found in VV-infected cells. A shorter time-course analysis of the extent of inhibition of protein synthesis and of eIF-2α-S51 phosphorylation indicates that such effects are clearly observed by 6 h.p.i in VT7-HCV7.9 infected cultures in the presence of IPTG (not shown).

**Figure 5:**
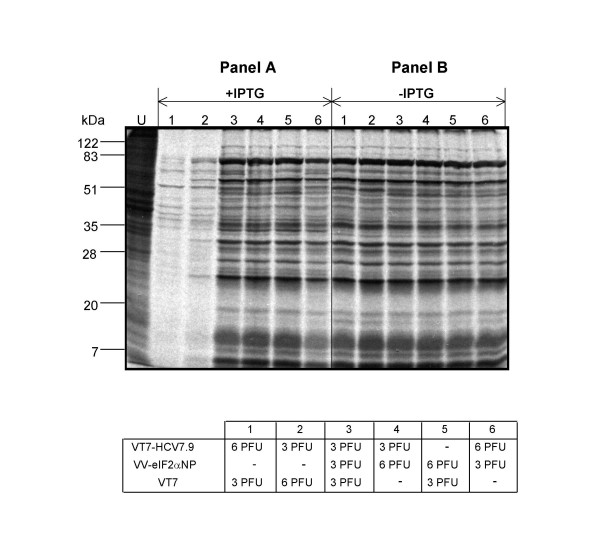
**Expression of the dominant negative eIF-2α-S51A mutant by VV-eIF2αNP rescues the translation inhibition induced by HCV polyprotein**. BSC40 cells grown in 12-well plates were infected at a total of 9 PFU/cell with the viruses indicated in the presence or absence of IPTG (1.5 mM). At 18 h.p.i. the cells were metabolically labeled with [^35^S] Met-Cys Promix (50 μCi/mL) for 30 min. and analysed by SDS-PAGE (12%) and autoradiography.

To further assess the role of eIF-2α phosphorylation on the translational arrest, we examined whether expression of the dominant negative non-phosphorylated mutant Ser51-Ala (eIF-2α-S51A) was capable of rescuing the translation inhibitory effects of HCV gene expression. To this end, different combinations of recombinant viruses, VT7-HCV7.9, VT7 and VV-eIF2αNP (inducibly expressing the eIF-2α-S51A mutant), were assayed in the presence or absence of IPTG. The metabolic labelling of infected cells revealed that expression of eIF2α-S51A mutant in cells co-infected with VT7-HCV7.9 in the presence of IPTG, rescues the translational block caused after HCV polyprotein expression (Figure 5A: compare lanes 3, 4 and 6 with lanes 1 and 2). In the absence of IPTG, protein synthesis levels were not affected (Figure 5B).

The above findings demonstrate that the translational block induced after HCV polyprotein expression from VV involves eIF-2α phosphorylation.

### HCV polyprotein expression from VV in the hepatic cell line HepG2 inhibits cellular and viral protein synthesis

**Figure 6:**
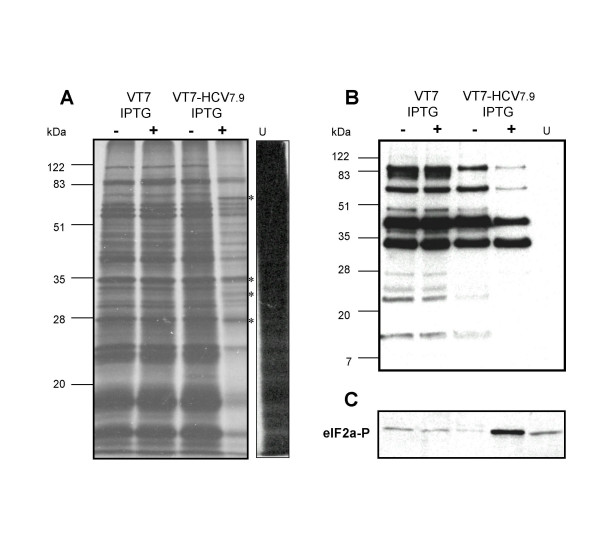
**Expression of HCV polyprotein from VV inhibits cellular and viral protein synthesis in the hepatic cell line HepG2**. **A**: Monolayers of HepG2 cells were infected (5 PFU/cell) with either VT7 or VT7-HCV7.9 recombinant viruses, in the presence (+) or absence (-) of the inductor IPTG. Uninfected (U) and infected cells were metabolically labelled with [^35^S] Met-Cys Promix (100 μCi/mL) from 4 to 24 h.p.i and treated as described under Materials and Methods. Approximately 100 μg of total cell protein extracted from uninfected and infected cells was fractionated by SDS-PAGE followed by autoradiography. (*) represents new additional polypeptides corresponding to the HCV proteins. **B**: Immunoblot analysis of total cell protein lysates prepared from uninfected and infected cells at 24 h.p.i. The blot was probed with a rabbit polyclonal anti-serum raised against live VV. **C**: The blot was stripped and probed again with a polyclonal antibody that recognized phospho-eIF-2α-S51 protein.

The HCV is a hepatotropic virus, thus we set out to study the effects of HCV gene expression in a hepatoblast cell line. HepG2 cells were infected with VT7 or VT7-HCV7.9 in the presence or absence of IPTG, metabolically labelled with ^35^S-Met-Cys Promix from 4 to 24 h.p.i, cell extracts fractionated by SDS-PAGE, and the protein pattern visualized upon autoradiography analysis. As shown in Figure 6A, cells infected with the recombinant VT7-HCV7.9 virus in the presence of IPTG demonstrated the synthesis of new additional polypeptides corresponding to HCV proteins (confirmed by Western blot, not shown), with a marked reduction in protein synthesis, in comparison with cells infected in the absence of the inductor, or in those cells inducibly expressing the T7 RNA polymerase (VT7). Expression of HCV results in decreased levels of VV proteins, as shown by a Western blot using anti-VV antibodies (Figure 6B) and increased phosphorylation levels of eIF-2α-S51 (Figure 6C). These results indicate that HCV polyprotein expression from VV inhibits cellular and viral protein synthesis in hepatoblast cells, which correlates with eIF-2α-S51 phosphorylation.

### Phosphorylation of eIF-2α and translational inhibition induced by HCV polyprotein expression from VV is mediated by PKR

Inhibition of translation through phosphorylation of eIF-2α, is a major stress-responsive checkpoint employed by at least four cellular kinases: PKR, PERK, GCN2, and HRI [24-27]. In particular of these four kinases, PKR has been shown to be the key regulator of cell defence against viral infections, and mediates the antiviral and antiproliferative effects of interferon (IFN) [28]. Activated PKR phosphorylates the α subunit of eIF-2 on serine 51, thus halting initiation of translation of both cellular and viral proteins that eventually leads to inhibition of viral replication [24].

**Figure 7:**
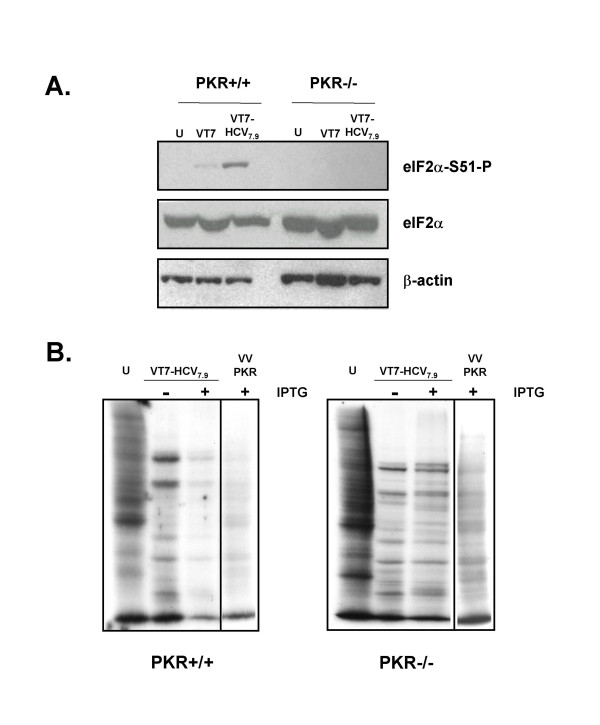
**PKR mediates phosphorylation of eIF-2α and inhibition of translation caused by the expression of HCV polyprotein**. **A**: Immunoblot analysis of total cell protein lysates prepared from PKR knockout (PKR-/-) and PKR WT (PKR+/+) cells infected with the parental (VT7) or the recombinant VT7-HCV7.9 viruses in the presence (+) of IPTG for 24 h. The blot was first probed with a polyclonal antibody that recognized phospho-eIF-2α-S51 protein, stripped twice, and reprobed with a polyclonal antibody that recognizes total eIF-2α protein and a monoclonal antibody against β-actin. **B**: Wild type and PKR-/- cell lines infected with VT7-HCV7.9 in the presence (+) or absence (-) of IPTG were metabolically labelled with ^35^S-Met-Cys Promix (50 μCi/mL) at 16 h.p.i, fractionated by SDS-PAGE and analysed by autoradiography. The recombinant VV-PKR virus was used as a control. U: uninfected cells.

In order to determine if PKR was the kinase responsible for eIF-2α phosphorylation following expression of HCV from VV, we infected PKR knockout cells (PKR-/-) and PKR WT cells (PKR+/+) with VT7 or VT7-HCV7.9 recombinant viruses in the presence of IPTG. As shown in Figure 7A, higher eIF-2α phosphorylation levels were observed in PKR+/+ than in PKR-/- cells after VT7-HCV7.9 infection. The total levels of eIF-2α and β-actin proteins were similar for both cell lines, in uninfected, as well as in VT7 or VT7-HCV7.9 infected cells. To corroborate whether eIF-2α phosphorylation halts translation of cellular and viral proteins, PKR-/- and PKR+/+ cells were infected with VT7-HCV7.9 in the presence or absence of IPTG, metabolically labelled, cell extracts fractionated by SDS-PAGE and proteins pattern visualized employing autoradiography. Only those PKR+/+ VT7-HCV7.9 infected cells in the presence of IPTG, showed a significant reduction of cellular and viral protein synthesis (Figure 7B). As expected, the expression of PKR by VV-PKR when used as a positive control, suppressed protein synthesis in both cell lines. Those data indicates that such cells are responsive to exogenous PKR delivered by VV.

These findings reveal that PKR is the kinase responsible for eIF-2α phosphorylation as well as for the translational block following HCV polyprotein expression from VV in infected cells.

### HCV polyprotein expression from VV induces apoptosis in HeLa and HepG2 cells, an effect that is caspase-dependent

**Figure 8:**
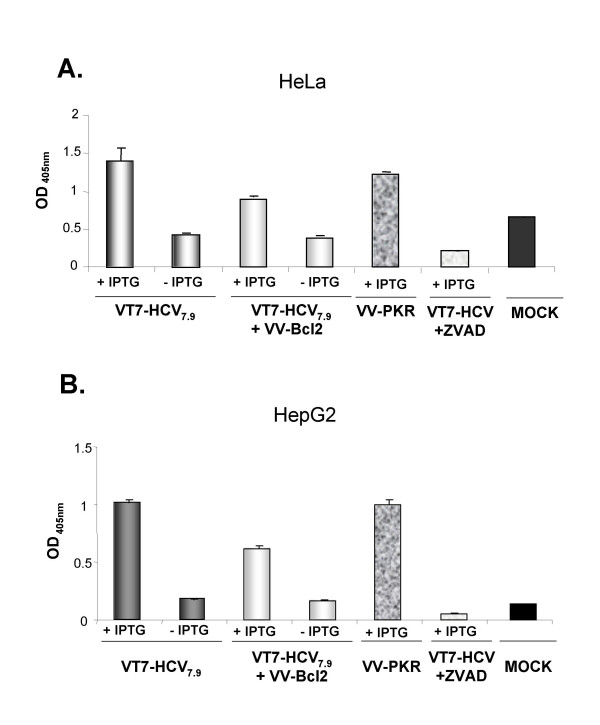
**Expression of HCV polyprotein from VV induces apoptosis in HeLa and HepG2 cells that is caspase-dependent**. **A**: HeLa cells were infected at 5 PFU/cell with the recombinant VT7-HCV7.9 individually or in combination (2.5 PFU of each virus/cell) with the recombinant VV-Bcl2 (inducibly expressing the anti-apoptotic Bcl-2 polypeptide) or with a general caspase inhibitor, Z-VAD-FMK (Calbiochem) at 50 μM, in the presence (+) or absence (-) of IPTG. The apoptotic levels were determined at 24 h.p.i by ELISA. **B**: HepG2 cells were infected at 10 PFU/cell with the recombinant VT7-HCV7.9 individually or in combination (5 PFU of each virus/cell) with the recombinant VV-Bcl2 or with a general caspase inhibitor, Z-VAD-FMK (Calbiochem) at 50 μM, in the presence (+) or absence (-) of IPTG. The apoptotic levels were determined at 48 h.p.i by ELISA. VV-PKR infected cells in the presence (+) of IPTG were used as positive controls.

It has been reported that expression in hepatic cells of all structural and nonstructural proteins from HCV cDNA [29] or from full-length RNA [30], can lead to apoptotic cell death, which may be an important event in the pathogenesis of chronic HCV infection in humans. To investigate whether apoptosis occurs in our virus-cell system, HeLa and HepG2 cells were infected with the recombinant VT7-HCV7.9 or coinfected with the recombinant VV-Bcl2 (that inducibly expresses the anti-apoptotic Bcl-2 polypeptide) in the presence or absence of IPTG. The levels of apoptosis were determined at 24 h.p.i (for HeLa cells) or at 48 h.p.i (for HepG2 cells), using an ELISA-based assay that detects the amount of cytoplasmic histone-associated DNA fragments. As shown in Figure 8 (panels A and B), expression of HCV by VT7-HCV7.9 in the presence of IPTG, induces apoptosis to levels similar to those obtained in induced VV-PKR-infected cells, used as a positive control. These apoptosis levels were two fold higher than those found in uninduced VT7-HCV7.9 infected cells. Co-expression from VV of HCV and of Bcl-2 in HeLa and HepG2 cells infected in the presence of IPTG, generates a two-fold reduction in apoptosis levels. A higher reduction in apoptosis was obtained by the Z-VAD-FMK general caspase inhibitor. These results revealed that HCV polyprotein expression from VV induced an apoptotic response, an effect mediated by caspases.

### Apoptosis induced by HCV polyprotein expression from VV is mediated by RNase L in a PKR-independent manner

In addition to PKR, the antiviral effects of IFN are executed through the functions of various proteins, including 2'5 oligoadenylate synthetase (2'-5AS), RNase L and Mx [31-34]. The 2'-5AS/RNase L and PKR pathways respond to dsRNA produced during the course of viral infections, to trigger an antiviral response in cells through RNA degradation and inhibition of protein synthesis. In contrast, Mx proteins obstruct the replicative cycles of particular negative strand RNA viruses by interfering with the intracellular movement and functions of viral proteins [28].

**Figure 9:**
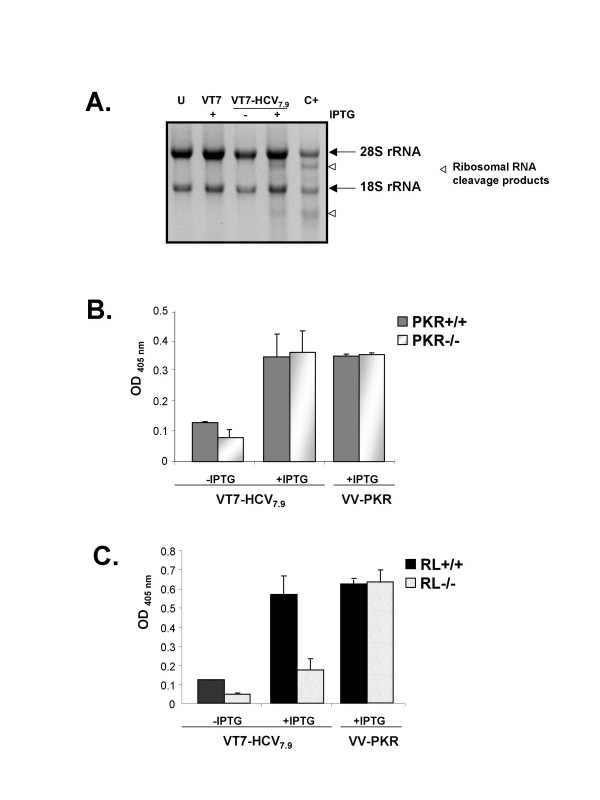
**Expression of HCV polyprotein from VV induces ribosomal RNA degradation mediated by RNaseL and triggers apoptosis through RNase L independently of PKR**. **A**: Monolayers of HeLa cells were either uninfected (U), single-infected with VT7 (5 PFU/cell), single-infected with VT7-HCV7.9 (5 PFU/cell) in the presence (+) or absence (-) of IPTG, or triple-infected with VV-RL + VT7 + VV-25AS (2 PFU of each virus/cell) (C+). Infections proceeded for 24 hours. 2 μg of total RNA was fractionated in 1% agarose-formaldehyde gel and stained with ethidium bromide. Abundant ribosomal RNAs 28S and 18S are indicated. **B and C**: PKR knockout (PKR-/-) and PKR WT cells (PKR+/+) (panel B), as well as RNase L knockout (RL-/-) and RNase L WT cells (RL+/+) (panel C), were infected at 5 PFU/cell with the recombinant VT7-HCV7.9 virus, in the presence (+) or absence (-) of the inductor IPTG. The apoptotic levels in cell extracts were determined at 24 h.p.i. by ELISA. The recombinant VV-PKR virus was used as a control. U: Uninfected cells.

Once it was verified that PKR was the kinase responsible for eIF-2α phosphorylation and for the translational block following expression of HCV from VV, we assayed the activity of RNase L under the same conditions. HeLa cells were infected with VT7 or VT7-HCV7.9 recombinants in the presence or absence of IPTG for 24 h. Total RNA was fractionated in 1% agarose-formaldehyde gel and stained with ethidium bromide. As shown in Figure 9A, cells infected with VT7-HCV7.9 in the presence of IPTG exhibited ribosomal RNA degradation. This effect is mediated by RNase L since a similar pattern of rRNA cleavage products is observed by the co-expression of RNase L and 2-5AS delivered by the recombinant VVs, used as a positive control. In cells infected with either VT7 or VT7-HCV7.9 in the absence of IPTG, ribosomal RNAs were intact. The results of Figure 9A reveal that expression of HCV from VV induces the activation of RNase L.

One interesting parallel between the PKR and 2-5A system is that both pathways contribute to apoptosis [35,36]. In order to compare the role of these pathways in the apoptotic response induced by HCV, we used PKR and RNase L knockout cells. PKR+/+ and PKR-/- as well as RL+/+ and RL-/- cells were infected with VT7 or VT7-HCV7.9 recombinants in the presence of IPTG, and the apoptotic levels were determined by ELISA at 24 h.p.i. As seen in Figure 9, expression of HCV by VT7-HCV7.9 induces apoptosis in PKR+/+ (Figure 9B) and RL+/+ cells (Figure 9C). The levels of apoptosis were similar to those obtained after the expression of PKR from VV-PKR, used as positive control. The levels of apoptosis induced by VT7-HCV7.9 after addition of IPTG, were significantly decreased in RL-/- infected cells (Figure 9C), while in PKR-/- cells, such levels remained similar to those in PKR+/+ cells (Figure 9B). These findings indicate that expression of HCV by VT7-HCV7.9 triggers apoptosis through RNase L, in a PKR-independent pathway.

**Figure 10:**
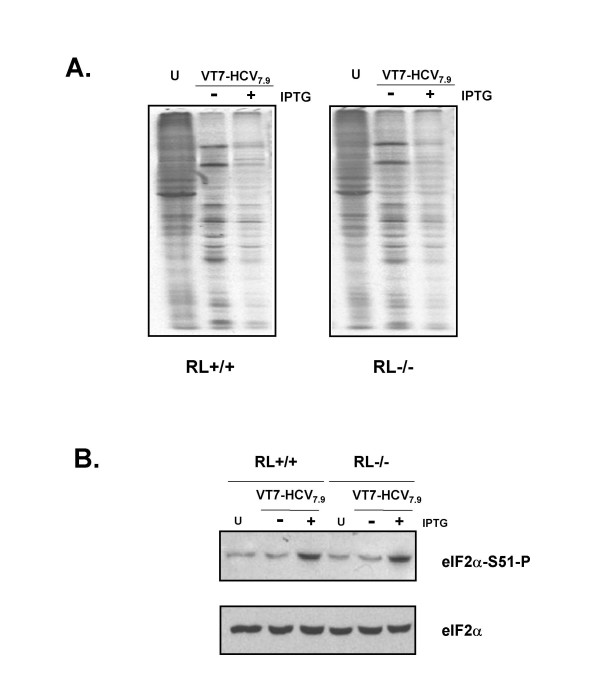
**Expression of HCV polyprotein from VV inhibits cellular and viral protein synthesis in RL+/+ and in RL-/- infected cells**. **A**: RL+/+ and RL-/- cells infected with VT7-HCV7.9 in the presence (+) or absence (-) of IPTG were metabolically labelled with ^35^S-Met-Cys Promix (50 μCi/mL) at 8 h.p.i, fractionated by SDS-PAGE and analysed by autoradiography. U: uninfected cells. **B**: Immunoblot analysis of total cell protein lysates prepared from RL+/+ and RL-/- cells infected with VT7-HCV7.9 in the presence (+) or absence (-) of IPTG for 8 h. The blot was first probed with a polyclonal antibody that recognized phospho-eIF-2α-S51 protein, stripped and reprobed with a polyclonal antibody that recognizes total eIF-2α protein.

Finally, we analysed cellular and viral protein synthesis in RNase L knockout cells expressing HCV. Consequently, RL+/+ and RL-/- cells were infected with VT7-HCV7.9 in the presence or absence of IPTG, metabolically labelled, cell extracts fractionated by SDS-PAGE and the pattern of proteins visualized using autoradiography. As shown in Figure 10, the expression of HCV provokes a similar reduction of cellular and viral protein synthesis in RL-/- and RL+/+ infected cells upon induction with IPTG (Figure 10A). This translational block correlates with increased levels of phosphorylation eIF-2α-S51 (Figure 10B) through PKR which is active in both cell lines. This result corroborates that apoptosis induced by HCV through RNase L is independent of the inhibition of protein synthesis caused by PKR.

## Discussion

Understanding the molecular mechanisms by which IFN-based therapies decreases HCV viral load, reduces the number of viral quasispecies, improves liver function, and reduces liver fibrosis in 15–30% of patients, is a priority in HCV research. Consequently, both viral and host factors have been implicated during the effective clinical response or resistance phenomenon of patients to IFN treatment [37]. Different *in vitro *model systems have been developed to study the role of HCV polyprotein on host cell responses [12-21]. The implication of IFN-induced genes and their action in the antiviral response of the host to HCV expression is not yet fully understood.

To further characterize the antiviral response of the host during expression of HCV polyprotein, we developed a novel virus-cell system based on a poxvirus vector, that inducibly expresses the structural and nonstructural (except part of NS5B) proteins of HCV ORF from genotype 1b. The generated recombinant VT7-HCV7.9 virus contains the HCV DNA coding region inserted within the VV HA locus, under the transcriptional control of a T7 promoter, and expresses the T7 RNA polymerase upon induction with IPTG (see Figure 1A). Current systems relying on viral delivery of T7 RNA polymerase are restricted by the efficiency with which HCV cDNAs can be transfected into cells, which in the case of hepatocyte and hepatocyte-derived cell lines, is often low [16-18]. The poxvirus-based system described here permits both the regulated production of the HCV transcripts into cells and the efficient delivery of the HCV genome into a wide variety of primary and continuous cell lines.

In this study, we demonstrate that upon induction with IPTG, HCV proteins are efficiently produced in VT7-HCV7.9 infected cells of various origins. This observation indicates that the DNA fragment of HCV ORF included in the VV genome, is efficiently transcribed and translated into a viral polyprotein precursor that is correctly processed into mature structural and nonstructural HCV proteins, as confirmed with specific antibodies to individual HCV proteins. Significantly, inducible expression of HCV polyprotein in VT7-HCV7.9 infected cells caused a considerable reduction in the production of infectious VV, as well as striking inhibition in total protein synthesis, both viral and cellular. The translational block was observed by 6 h.p.i when all of the HCV proteins were produced. The inhibition of protein synthesis by HCV was highly specific and could not be solely attributed to the induction of HCV RNA transcript since cells infected with VT7-VP3 that expressed the IBDV ORF VP3 mRNA, did not trigger translational inhibition. Furthermore, the HCV ORF included in the VT7-HCV7.9 recombinant virus lacks the 5' UTR, bearing the HCV IRES, and the 3' UTR, both implicated in HCV replication and liver injury [38]. The inhibition of protein synthesis that we have observed in induced VT7-HCV7.9 infected HeLa and HepG2 cells was associated with a significant increase in the phospho-eIF-2α-S51 levels, suggesting that HCV expression might control the cellular translation through eIF-2α-S51 phosphorylation. This translational control was confirmed with a dominant negative non-phosphorylated (NP) mutant Ser51-Ala (eIF-2α-S51A). Expression of the eIF-2α-S51A mutant in cells co-infected with VV-eIF-2α-NP and VT7-HCV7.9 in the presence of IPTG, rescued the translational block induced by HCV (Figure 6). Moreover, we showed that phosphorylation of eIF-2α-S51 was carried out by the cellular kinase PKR, as revealed in knockout PKR-/- cells (Figure 9). The role of PKR and eIF-2α-S51 phosphorylation in HCV infection has been widely studied due to the relevance of this kinase in the cellular antiviral response. As has been previously reported [23,39-41], PKR mediated phosphorylation of eIF-2α-S51 results in inhibition of translation and a blockade of viral protein synthesis, which in turn, inhibits virus replication. For this reason, viruses employ a variety of strategies to inhibit PKR activation and function. Several groups have described the role of certain HCV proteins in cellular translation. HCV NS4A and NS4B proteins mediate translational inhibition and, perhaps, increased degradation of certain cellular proteins [42,43]. In contrast, NS5A and E2 proteins are reported to enhance translation by inhibiting PKR functions [44,45]. Therefore, it seems that during the course of HCV infection, there is a balance between inhibition and enhancement of host cell translation depending on the degree of activation/inhibition of the PKR pathway. Most of these studies have relied on systems that express HCV proteins individually. Nontheless, since all HCV proteins are potentially produced *in vivo *during virus infection of hepatocytes, it is important to use a full-length genome rather than individual HCV proteins to study the molecular mechanisms involved in virus-host cell interactions and in HCV pathogenesis. In our viral delivery system, the overall expression of structural and nonstructural HCV proteins by recombinant VT7-HCV7.9 virus did not reverse the action of PKR, since host cell translation was inhibited through phosphorylation of eIF-2α-S51 by the kinase. An incapability to prevent PKR activation by HCV polyprotein expression was reported by François and co-workers when they analysed the response to IFN of the human cell line UHCV-11 engineered to inducibly express the entire HCV genotype 1a polyprotein [46]. Although we could not exclude the possibility that a certain level of inhibition of PKR by NS5A or E2 occurs at a much localized level, the resistance to IFN exhibited by some HCV genotypes as a result of viral protein expression, cannot be explained solely by inhibition of the negative control of PKR translation. It is possible that during the course of HCV infection, NS5A plays a role in inhibiting PKR locally at the site of HCV protein synthesis. NS5A may, however, participate in the blockade of IFN's antiviral action through another mechanism, such as the reported interaction with the Ras-associated Grb-2 protein [47]. These results confirm the necessity to re-evaluate all types of interactions between any particular HCV protein and its cellular partner(s) in the context of expression of all of the HCV proteins. Consequently, as shown here by confocal microscopy (Figure 2), the HCV proteins are localized within aggregates in the cell cytoplasm which might influence their interaction with PKR, a protein found surrounding the nucleus, in microsomes and in the nucleolus [24,48].

Several *in vitro *studies reveal that synthesis of HCV structural proteins or the full-length genome have a direct cytotoxic effect or activate an apoptotic response in osteosarcoma, hepatoma and B cell lines [29,30,49-51]. Furthermore, the alteration of ER membranes [52] and the activation of signalling pathways characteristic of an ER-stress condition, have been found to be associated with the expression of HCV proteins [53-55]. Although these data suggest that HCV may alter intracellular events with possible consequences on liver pathogenesis, the complex mechanism and the role of the viral proteins implicated are currently unknown. As we have shown in this work, expression of most of the HCV genome from VV induces a cell death phenomenon by apoptosis that should contribute to liver pathogenesis. Apoptosis induced by HCV polyprotein expression was prevented by Bcl-2 and by a general caspase inhibitor (Z-VAD-FMK) indicating a caspase-dependent death process. Even though PKR is the main kinase responsible for eIF-2α phosphorylation and for translation inhibition induced by the expression of HCV in VT7-HCV7.9 infected cells, it does not appear to be involved in apoptosis within this system, as revealed from studies performed in PKR+/+ and PKR-/- knockout cells. The extent of apoptosis induction by HCV expression was the same in PKR+/+ and in PKR-/- cells (Figure 9B), suggesting that other pathways may be involved. PKR induces apoptosis in response to activation by different stimuli, such as the accumulation of dsRNA as a by-product during virus replication [36], or when PKR is overexpressed in cells [56]. Several authors, however, have reported that PKR can also be activated through the binding of heparin and other polyanions [57,58], or by the cellular activator protein PACT/RAX [59,60]. The events that mediate induction of apoptosis by PKR have been widely studied and both PKR-induced translational block by phosphorylation of eIF-2α, and NF-kB activation, have been shown to be activated during apoptosis [61]. Since PKR has a number of potential substrates and signalling targets, it is likely that the phosphorylation of eIF-2α by PKR in response to HCV expression is not sufficient to mediate the pro-apoptotic effects of this kinase.

In this study, we also demonstrate the activation of endogenous RNase L and its role in the apoptosis induced by HCV expression (Figure 9A,C). Although it is widely accepted that the IFN-induced proteins PKR and RNase L require the expression of dsRNA for their activation (either directly in the case of PKR or indirectly via 2'-5'-OAS in the case of RNase L), there are several reports that documented the effect of HCV proteins on PKR and 2'-5'-OAS activation. The NS5A and E2 proteins can suppress the PKR pathway [44,45], whereas the Core protein can transcriptionally activate the 2'-5'-OAS gene through an IRES present within IFN-inducible gene promoter [62]. Like PKR, the 2-5AS/RNase L system can control virus growth by inducing apoptosis in response to viral infection [35,36]. Overexpression of RNase L or activation of the endogenous enzyme induces apoptosis by a mitochondrial-caspase dependent pathway that is suppressed by Bcl-2 [63-65]. Similarly, apoptosis induced by HCV polyprotein expression was inhibited by Bcl-2 (Figure 8). Although the apoptotic levels induced by HCV proteins remain invariable in PKR+/+, PKR-/-, and RL+/+ cells, the levels are significantly decreased in RL-/- cells, indicating that inducible expression of HCV proteins by VT7-HCV7.9 triggers apoptosis through RNase L in a PKR-independent pathway. Under physiologic conditions, RNase L activity is tightly regulated by 2'-phosphodiesterase and RNase L inhibitor [66,67] such that only a limited activation of RNase L occurs. The mechanism of the regulation of RNase L inhibitor is unknown, but the reduction of its expression seems to be advantageous for host defence together with the enhanced 2-5 OAS activity. Yu and co-workers [68] described that hepatic overexpression of PKR mRNA, and reduced expression of an RNase L inhibitor mRNA, are parameters that seem to contribute to an anti-HCV response. In agreement with our results, it has been reported that the absence of RNase L has an anti-apoptotic effect in multiple cell types treated with a variety of different agents [69]. The effects that have been observed in this study upon HCV polyprotein expression from VV are likely to have biological significance during HCV infection as there is ample evidence that VV recombinants can be used to study the function of multiple genes and that the assigned function mimics the effects described in non-viral systems [70].

## Conclusion

We have developed an efficient viral delivery system expressing the polyprotein of HCV in numerous mammalian cell lines in a faithfully, efficient and time regulated manner, allowing us to analyze the host response to HCV proteins. We demonstrate that two components of the interferon (IFN) system, protein kinase PKR and RNase L, are activated during HCV polyprotein expression and are responsible for translational control and induction of apoptosis. These two pathways are likely to limit the replication capacity of HCV. Thus, the virus-cell system described here highlights the relevance of the IFN system as a protective mechanism against HCV infection.

## Methods

### Cells and viruses

Cells were maintained in a humidified air 5% CO_2 _atmosphere at 37°C. African green monkey kidney cells (BSC40) and human cells (HeLa) were grown in Dulbecco's modified Eagle's medium (DMEM) supplemented with 10% newborn calf serum (NCS). Human HepG2 hepatocellular carcinoma cells (ATCC HB-8065) were maintained in DMEM supplemented with penicillin (0.6 μg/mL); streptomycin (60 μg/mL); glutamine (2 mM); N-2-hydroxyethylpiperazine-N'-2-ethanosulfonic acid (HEPES) buffer, pH 7.4 (20 mM) and 10% fetal calf serum (FCS). Mouse 3T3-like fibroblasts derived either from homozygous PKR knockout mice (PKR-/-) or PKR wild type mice (PKR+/+) [71] were obtained from C. Weissmann (University of Zurich, Switzerland) and grown in DMEM supplemented with 10% FCS. Wild type mouse embryo fibroblasts (MEFs) derived from C57BL6 mice (RL+/+) and fibroblast lacking the RNase L gene (RL-/- MEFs) derived from mice with the RNase L gene disrupted [35], were propagated in DMEM supplemented with 10% FCS, and were a gift from R. Silverman (Cleveland Clinic, USA)

The recombinant vaccinia virus (VV) that is inducible and expresses the T7 RNA polymerase (VT7lacOI) was previously described [72]. Virus VT7-VP3 expressing the IBDV mature structural capsid protein VP3 [73] was kindly provided by J.F. Rodríguez (CNB, Spain). VVeIF-2α NP was generated through homologous recombination in TK^- ^143B cells, as previously reported [74]. The recombinant VV-PKR TK^- ^expressing IPTG-inducible PKR was generated by homologous recombination of their respective pPR35-derived plasmid with the WR strain of VV in BSC40 cells, as previously described [56]. VV recombinant expressing Bcl-2 protein (VV-Bcl2) was generated as previously reported [75]. The recombinant vaccinia viruses VV-RL and VV-2-5AS were obtained after introduction of plasmid pTM-RL and pSC-2-5AS respectively into the TK region of wild-type vaccinia virus (WR) DNA by homologous recombination as described [76]. All VV recombinants were grown in BSC40 cells and purified by banding on sucrose gradients [77].

### Generation of the recombinant vaccinia VT7-HCV7.9 virus

A 7.9 Kb DNA fragment containing the structural (C, E1, E2 and p7) and nonstructural (NS2, NS3, NS4A, NS4B, NS5A and the amino terminal region of NS5B) proteins of HCV ORF from genotype 1b was excised with *EcoRI *from the original full-length HCV genome containing plasmid pcDNA-hcv1b (kindly provided by Ilkka Julkunen from National Public Health Institute, Finland). This DNA fragment was cloned into the VV insertion/expression vector pVOTE.1 [72] previously digested with *EcoRI *and dephosphorylated by incubation with alkaline phosphatase, Calf Intestinal (CIP) as described in Figure 1A. The resulting plasmid, pVOTE.1-HCV7.9 directs the insertion of HCV genes into the HA locus of the VT7lacOI genome under the transcriptional control of the T7 promoter. BSC40 cells were infected with the recombinant vaccinia virus VT7lacOI at a multiplicity of 0.05 PFU/cell, and then transfected with 10 μg of plasmid DNA pVOTE.1-HCV7.9 using lipofectamine reagent according to manufacturer's instructions (Invitrogen). The selection and amplification of the recombinant VT7-HCV7.9 virus was carried out as previously described [78]. The purity of the recombinant virus was confirmed by PCR analysis. The plasmid pVOTE.1 as well as the VV recombinant VT7lacOI, were kindly provided by Bernard Moss (NIH, USA).

### Metabolic labelling of proteins

Different cell lines grown in 12 well plates were infected at an infection multiplicity of 5 PFU/cell with the viruses indicated, and maintained either in the presence or absence of the inductor isopropyl-β-D-thiogalactoside (IPTG) (1.5 mM final concentration). For continuous metabolic labelling of proteins, the cells were rinsed three times with Met-Cys-free DMEM at 4 h post-infection (p.i) and incubated with 100 μCi of [^35^S] Met-Cys Promix (Amersham) per mL in a mixture of Met-Cys-free DMEM and complete DMEM (9:1) for 16–20 h. After three washes with phosphate buffered saline (PBS) cells were resuspended in Laemmli buffer and analysed by sodium-dodecyl sulfate-polyacrylamide gel electrophoresis (SDS-PAGE) followed by autoradiography. For discontinuous metabolic labelling of proteins, the cells were rinsed three times and incubated with Met-Cys-free DMEM 30 minutes prior to labelling. After incubation, the medium was removed and 50 μCi of [^35^S] Met-Cys Promix per mL in Met-Cys-free DMEM was added for an additional 30 minutes. The cells were washed with PBS and treated as described above.

### Immunoblotting

The HCV-antibody positive human sera used in this study was kindly provided by Dr Rafael Fernández from the Ramón and Cajal Hospital (Spain). The rabbit polyclonal anti-serum against live vaccinia virus was previously described [79]. The rabbit polyclonal anti eIF2α [PS^51^] phosphospecific antibody was supplied by BIOSOURCE. The monoclonal antibody against β-actin was supplied by SIGMA. Rabbit polyclonal anti-eIF2α antibody was supplied by Santa Cruz, CA.

For immunoblot analyses, total cell extracts were boiled in Laemmli sample buffer, and proteins were fractionated by 12% SDS-PAGE. After electrophoresis, proteins were transferred to nitrocellulose membranes using a semi-dry blotting apparatus (Gelman Sciences). Filters were mixed with antisera in PBS containing non-fat dry milk at 5% (BLOTTO), incubated overnight at 4°C, washed three times with PBS, and further incubated with secondary antibody coupled to horseradish peroxidase in BLOTTO. After the PBS wash, the immunocomplexes were detected by enhanced chemiluminescense Western blotting reagents (ECL) (Amersham).

### Immunofluorescence

Specific antibody for Golgi apparatus (anti-Gigantine) was kindly provided by Manfred Renz from the Institute of Immunology and Genetics Karlsruhe (Germany).

HeLa cells cultured on coverslips were infected at 5 PFU/cell with VT7-HCV7.9 in the presence or absence of IPTG (1.5 mM final concentration). At 16 h.p.i, cells were washed with PBS, fixed with 4% paraformaldehyde and permeabilized with 2% Triton X-100 in PBS (room temperature, 5 min). Cells were incubated with a human antibody recognizing HCV proteins together with anti-Gigantine antibody. Coverslips were then extensively washed with PBS, and incubated in darkness for 1 h at 37°C, with secondary antibody conjugated with green fluorochrome Cy2 (Jackson Immunoresearch) and with the DNA staining reagent ToPro (Molecular Probes). Images were obtained by using Bio-Rad Radiance 2100 confocal laser microscope, were collected by using Lasersharp 2000 software and were processed in LaserPix.

### Measurement of the extent of apoptosis

The cell death detection enzyme-linked immunosorbent assay (ELISA) kit (Roche) was used according to manufacturer's instructions. This assay is based on the quantitative sandwich enzyme immunoassay principle, and uses mouse monoclonal antibodies directed against DNA and histones to estimate the amount of cytoplasmic histone-associated DNA fragments.

### Total RNA isolation

Total RNA from uninfected or infected cells was isolated using Ultraspect-II resin purification system (Biotecx). RNA was denatured and analyzed in 1% formaldehyde agarose gels and stained using ethidium bromide as previously described [76].

## Competing interests

The author(s) declare that they have no competing interests.

## Authors' contributions

CEG has generated the vaccinia virus recombinant VT7-HCV7.9 and has analyzed protein expression in culture cells. AMV has performed confocal microscopy and defined apoptosis in infected cells. MAG has performed PKR and RNase L assays with KO cells. EDG has performed rRNA cleavage assays. ME conceived the study, has supervised the work, and provided the tools necessary for the performance of the research.
